# Association of a kinematic measure of upper limb reaching and StartReact in chronic hemiparetic stroke

**DOI:** 10.1007/s00221-025-07047-2

**Published:** 2025-05-06

**Authors:** Supriyo Choudhury, Anna Baines, Swagata Sarkar, Asit Baran Bayen, Subhajit Sarkar, Mark R. Baker, Hrishikesh Kumar, Stuart N. Baker

**Affiliations:** 1 Department of Neurology, Institute of Neurosciences Kolkata, Kolkata, India; 2 Department of Physiology, University of Calcutta, Kolkata, India; 3 Faculty of Medical Sciences, Newcastle University, Newcastle upon Tyne, UK; 4 Department of Neurology, Royal Victoria Infirmary, Newcastle upon Tyne, UK; 5 Department of Clinical Neurophysiology, Royal Victoria Infirmary, Newcastle, UK

**Keywords:** Reticulospinal tract, Marker less computer vision technology, Upper limb reaching, StartReact, ARAT, DEEPLABCUT

## Abstract

**Background:**

After stroke, recovery of upper limb reaching movements may partly depend on the level of activation of the reticulospinal tract (RST), but few clinical studies have explored this. Here we examined the association between the strength of reticulospinal connections and extent of reaching in post-stroke patients.

**Methods:**

Fifteen patients (all male) with right hemiparesis who had suffered a stroke at least six months prior to the assessment were selected based on predefined selection criteria. Video recordings of the reaching task from the Action Research Arm Test (ARAT) were processed for two-dimensional kinematic analysis using the markerless motion tracking software DeepLabCut. We defined a novel Index of Elbow Extension (IoEE), and validated it by comparison between simultaneously obtained two- and three-dimensional datasets in healthy people. Strength of reticulospinal outputs was estimated using the ‘StartReact’ paradigm, which measures the speeding up of reaction time by a loud sound cue.

**Results:**

We observed a significant negative correlation between the IoEE and StartReact (rho = 0.9, *p* < 0.05). There was no correlation in this cohort between ARAT and StartReact.

**Conclusion:**

We speculate that the negative correlation between reaching performance and StartReact is a consequence of the variable compensatory activation of the reticulospinal tract (RST) in response to different levels of initial damage. This study reinforces the application of freely available computer vision technology for assessment of two- dimensional kinematics in a clinical scenario.

## Introduction

Recovery of upper limb function after stroke results from plasticity in cortical and subcortical neural connections (Baker 2011; Zaaimi et al. 2012). The reticulospinal tract (RST) is a primitive motor tract originating from the pontomedullary reticular formation (PMRF). In humans recovering from hemiparetic stroke, reticulospinal pathways are upregulated during motor recovery (Choudhury et al. 2019). To date, most studies in this field have implemented electrophysiological and anatomical methods in animal models of stroke (Buford and Davidson 2004; Davidson and Buford 2006; Zaaimi et al. 2012, 2018; Herbert et al. 2015; Plautz et al. 2023), whereas reports exploring the relation between reorganization of the RST and motor recovery in humans are relatively limited (Choudhury et al. 2019; Rahimi and Honeycutt 2020).

The complex process of upper limb motor recovery after hemiparetic stroke is likely to be determined by multiple physiological processes, which may or may not be interdependent, including individuation, spasticity, synergies and strength (Zackowski et al. 2004). These will all impact on the functional outcome, providing varied restoration of motor function. Functional recovery depends on regaining important abilities such as reaching, power grip, pinch grip and lifting capacity (Zackowski et al. 2004). Skilled reaching is one of the most primitive movements from an evolutionary perspective, and key to survival in many animals (Sacrey et al. 2009). This can be quantified objectively by assessing kinematic parameters of the reach (Zackowski et al. 2004). Whilst axial and proximal limb muscles may have a strong influence on reaching movements, for humans, extension around the elbow is equally important.

In contrast to the more classic view, which frames the RST as a controller of tone in axial and limb girdle muscles, recent studies have also demonstrated its influence on both proximal and distal upper limb muscles (Davidson and Buford 2006; Riddle et al. 2009; Honeycutt et al. 2013; Baker and Perez 2017). The RST can control both flexor and extensor muscles: ipsilateral RST projections tend to activate flexors, whereas contralateral projections favor extensors (Davidson and Buford 2006). Neurons in the reticular formation discharge with reaching movements (Schepens and Drew 2004) and are tuned to movement direction (Buford and Davidson 2004). In health, the control of reaching requires a coordination of outputs from both the RST and corticospinal tract (CST). While the RST appears best suited to control of commonly-used muscle patterns, the CST adds the ability to fractionate individual muscle activation (Zaaimi et al. 2018). The majority of descending drive to motoneurons during reaching movements appears to come from the RST (Tapia et al. 2022). After a stroke leading to loss of CST fibers, it might be hypothesized that the role of the RST would become even more dominant.

In humans there is only very limited and indirect evidence supporting a role for the RST in reaching (Glover and Baker 2019; Kearsley et al. 2022). Moreover, evidence from studies in patients recovering from hemiparetic stroke is mixed. For example, in one study, evidence of an increase in the strength of RST mediated reflexes in the affected arm was associated with adverse reaching outcomes (McPherson et al. 2018), whereas in other studies, electrophysiological measures of increased activity in RST pathways either had no relationship to the recovery of upper limb reaching (Hammerbeck et al. 2021) or were associated with increased reaching distance (Rahimi and Honeycutt 2020).

Recording physiological activity non-invasively from subcortical structures is challenging in humans. Startle, a brainstem-mediated motor reflex, is one method of probing RST function in humans (Davis et al. 1982). Another approach uses the ‘StartReact’ paradigm. Here, subjects respond rapidly to a visual cue with a movement, and the time to onset of EMG activity in a prime mover muscle is measured. On some trials, the visual cue is combined with a loud sound; this produces a speeding up of the reaction time. The extent of reaction time shortening appears to provide an estimate of the strength of reticulospinal connections (Smith et al. 2019; Tapia et al. 2022), and has been used in previous studies in both healthy human volunteers (Honeycutt et al. 2013; Maslovat et al. 2014; Dean and Baker 2017; Ossanna et al. 2019) and in patients with motor disorders (Fisher et al. 2013; Honeycutt et al. 2015; Baker and Perez 2017; Choudhury et al. 2019).

We recently observed a negative correlation between a clinical scale measuring upper limb function and StartReact in a large group of chronic stroke patients (Choudhury et al. 2019). Restoring upper limb functional capacity in stroke patients is inherently complex, as observed motor improvements are often affected by compensatory movements. Moreover, the score we used previously to evaluate upper limb function (Action Research Arm Test, ARAT) may place a disproportionate emphasis on hand function or distal motor control, rather than reaching. To address these limitations, in the current study we analysed archived video data captured during the ARAT assessment to determine whether a more objective marker of upper limb reaching is also correlated with RST activity in individuals with hemiparesis after stroke. While our primary goal was to investigate this neurobiological question, it also led to the validation of an inexpensive method for assessing upper limb kinematics using simple video recordings. We find that greater impairment of reaching is associated with increased RST connectivity, and suggest that more severe damage to cortical systems results in a greater reliance on sub-cortical systems.

## Methods

The data presented in this paper were collected as part of a previous study, the results of which have already been reported (Choudhury et al. 2019, 2020). The study was approved by the local Institutional Ethics Committee (INK/EthicsComm/46/2016) and conducted in compliance with the principles of the Declaration of Helsinki. Informed consent was obtained from all participants in writing before initiating any study procedures.

Patients were referred to the movement neuroscience laboratory from the Neurology and Neurorehabilitation out-patient department. Patients were not included if they had any type of aphasia, significant dementia, hearing or vision impairment, or fixed flexor deformities in the wrist joint. Individuals who had undergone botulinum toxin treatment for spasticity within the past three months were also excluded. Patients who had received a diagnosis of hemiparetic hemorrhagic or ischemic stroke, sparing the brainstem, at least six months prior to assessment were recruited to the study. Upper limb function was assessed using a validated rating scale, the Action Research Arm Test (ARAT) (Hsieh et al. 1998). The ARAT comprises four scoring domains (grasp, grip, pinch and gross) with a maximum possible cumulative score of 57 signifying minimal impairment. The maximum possible scores for the four subscales are: Grasp (18 points), Grip (12 points), Pinch (18 points), and Gross Movement (9 points). Each task within these subscales is rated on a 4-point scale, where 3 indicates normal performance, 2 reflects task completion with difficulty, 1 denotes partial completion, and 0 signifies an inability to complete the task. The final ARAT score is the sum of all subscale scores.

Kinematic data were extracted from video recordings acquired during completion of one task of the ARAT. Patients attempted to reach and grasp a small wooden cube (side 10 cm) placed on a table (height 75 cm) in front of them, and if able, to place this object on a shelf 50 cm higher than the table. The trunk was not restrained (e.g. by using straps to the backrest of the chair). This allowed subjects to bend forward while reaching if they wished, although this is not a typical behavior in individuals without reaching impairments. The hand started the reach in a comfortable resting posture; we did not attempt to impose a standardized starting position, but clearly instructed patients to reach without leaning forward if possible. The participants’ movements were monitored by an investigator. Each patient received verbal encouragement to complete the reaching task and was given multiple opportunities to succeed if they did not complete it on the first attempt.

Video recording was performed using a wall-mounted webcam (15 frames per second), approximately 1.5 m away from the chair on which the patient was seated, on the patient’s right side. Videos were first segmented to the reaching movement using start and finish times identified visually by an investigator (SC). Motion tracking of landmarks on the right upper limb (wrist, elbow, shoulder and nape of the neck) in each frame was performed using the open-source deep learning-based computer vision software DeepLabCut (DLC; version 2.0) (Mathis et al. 2018). Power grip strength was assessed with a Jamar-style electrodynamometer (G200, Biometrics Ltd, Newport, UK). Every patient was assessed three times with an interval of approximately 30 s and the largest grip force among three trials was used. The same protocol was repeated for the unaffected side. The grip strength of the affected side was normalized to that of the less affected side. Spasticity of the wrist flexor group of muscles was estimated using the modified Ashworth scale (MAS; Bohannon and Smith 1987).

We used historical control data on StartReact from 19 healthy participants (mean age 35 ± 11 years, 13 male), collected in the same setting and around the same time as the current study (Choudhury et al. 2019).

The StartReact protocol was used to assess the strength of RST connections. Participants placed their hand in between two adjustable plexiglass plates, with the semi-pronated forearm strapped to a rounded restraint. They were instructed to respond as quickly as possible to the flash of a light emitting diode (LED, 50ms flash duration) with an isometric wrist flexion. Three conditions were delivered in random order (*n* = 20 of each): LED alone; LED and low intensity sound (80 dB, 500 Hz, 50 ms duration) and LED plus high intensity, startling sound (110 dB, 500 Hz, 50ms duration), as used in our previous work (Dean and Baker 2017; Choudhury et al. 2019; Tapia et al. 2022). Surface EMG was recorded from the forearm flexor muscles using an EMG amplifier (gain 2 K, bandpass 30–2000 Hz), and digitized (sampling rate 5 kHz) using a 1401 laboratory interface (Cambridge Electronic Design, Cambridge, UK) interfaced with a personal computer running Spike2 software (also Cambridge Electronic Design, Cambridge, UK). Offline data analysis using a custom MATLAB script detected the EMG response latency for each trial. The latency was defined as the first deflection in the rectified EMG greater than the mean plus five times the standard deviation of a baseline period measured over the 200 ms before the LED flash. The MATLAB script provided for manual review of automatically detected response latencies, and manual correction if detection had been triggered spuriously, e.g. by electrical artifacts. The MATLAB program was used to generate the mean and standard deviations for Visual Reaction Time (VRT), Visual-Auditory Reaction Time (VART), and Visual Startle Reaction Time (VSRT). The difference between the mean response latency to low intensity sound (VART) and to high intensity sound (VSRT) was taken as the measure of the StartReact effect, which has been shown to relate to the strength of RST drive to motoneurons (Tapia et al. 2022).

Patients selected for inclusion in this study had to meet a number of criteria. The hemiparesis had to be on the right side (the same side as the fixed video camera); the hemiparetic limb had to be visible without obstructions throughout the video recording; and complete corresponding ARAT, grip strength and StartReact data sets had to be available. Patients were excluded where reaching was consistently suboptimal because of non-neural factors (e.g., arthritic pain, frozen shoulder or incorrect position of the shelf). We also excluded videos where clothing was likely to prevent accurate placement of markers. Initially, 20 frames were randomly selected by the DLC software to sample the manifold of reaching behavior for each patient. The operator then annotated the body parts in each frame manually, which served as training data for the deep learning network (ResNet-50, pretrained on ImageNet; Mathis et al. 2018). The trained network was applied to the entire video to produce a file giving the estimated pixel locations of each body part per frame. That output, without any additional filtering, was then processed through a MATLAB (MathWorks Inc, Natick, MA, USA) script to calculate an Index of Elbow Extension (IoEE) as follows:

$$\:IoEE=\:\frac{Maximum\:distance\:shoulder\:to\:wrist}{\left(Maximum\:distance\:shoulder\:to\:elbow\right)+\left(Maximum\:distance\:elbow\:to\:wrist\right)\:}$$

This measure was designed to assess the degree of elbow extension. It is bounded between zero (indicating full flexion at the elbow joint) and one (corresponding to full elbow extension). This approach had the advantage that it did not require the video measures to be converted from pixels into physical units (meters), because distances were normalized to generate a dimensionless quantity. In addition, the apparent length of limb segments will be distorted in these two- dimensional views by the angle between the camera and the patient’s body. However, the camera angle will affect each segment similarly and thus such distortions should be largely corrected by the normalization process. One limitation however is that the reaching distance was not determined based on the arm length of the subject. This could mean that variations in arm length could lead to small variations in IoEE, as people with longer arms would not need to extend the elbow as much to complete the task. The starting position for the reach was not rigidly enforced, although most subjects started with the hand on the lap. However, since the IoEE is related to the maximum elbow extension angle, the starting posture should have little effect; rather IoEE will be affected by the posture towards the end of the reach, when in a healthy person the elbow is extended.

In order to validate the IoEE determined as above, we gathered an extensive dataset in which healthy subjects mimicked the reaching movements of the stroke survivors. Subjects were seated before the ARAT test as described above. They were first shown a video of a patient performing a reach, and then asked to copy this movement. Their action was recorded by a Microsoft Azure Kinect DK camera operating at 30 frames per second. This device contains both a 12 megapixel color camera, and a 1 megapixel depth camera which records an estimate of the distance of the object away from the camera for each pixel. The two synchronized video streams were written to a single file. The color video was processed using DeepLabCut exactly as for the webcam-based recordings in stroke survivors, to extract 2D kinematics projected onto the plane of the camera; from this the IoEE was determined. The dual video stream was also processed through an artificial intelligence model developed by Microsoft (BodyTracking API), which generated the estimated location of 32 body segments in 3D; each coordinate was determined in millimeters from an origin point centered on the camera. A custom MATLAB program used the coordinates of the right shoulder, elbow and wrist to determine the elbow angle in degrees for each frame. The maximum elbow angle over the entire reach was then calculated. Twenty healthy subjects mimicked the movements of between four and six patients each, leading to a total of 102 videos. The correlation was then calculated between the IoEE (measured using 2D video) and maximum elbow angle (measured from 3D coordinates).

Statistical analysis was performed using MATLAB (v2022) and SPSS (2020, IBM inc.). The numerical data was reported as the mean ± standard deviation. The Spearman correlation was calculated between the IoEE and clinical measures; this was preferred over the Pearson’s correlation due to the relatively low sample size. For the larger dataset from healthy subjects used to validate IoEE, the Pearson’s correlation was used. Correction for multiple comparisons were made by the Benjamini-Hochberg procedure (Benjamini and Hochberg 1995), with a false discovery rate of 5%. The Benjamini-Hochberg procedure does not provide corrected P values; in the text and table, uncorrected values are given, together with a statement of whether these reached the significance threshold after multiple comparison correction.

## Results

### Patient cohort

Out of 95 patients recruited to the study, in 46 people usable StartReact data was available at the baseline visit. Among those 46 patients, 26 had right sided hemiparesis. Of these, four were excluded as the shelf was positioned too close to the patient, five were excluded because the bony landmarks were obstructed in some part of the video and two patients were completely unable to move the upper limb. This left videos from 15 people remaining for kinematic analysis. The mean age of the patients selected was 51 ± 12 years. Because clothing typically obscured video markers in the female patients, necessitating exclusion, only male patients were included in the final analysis. The mean ARAT score was 23.5 ± 18.9. The ARAT sub-scores were: Grasp, 7.9 ± 6.6; Grip, 5.2 ± 4.3; Pinch, 5.5 ± 6.9; Gross, 4.9 ± 2.0. The mean grip strength on the more affected side was 7.5 ± 5 kg (26.5 ± 8.8 kg on the less affected side). The normalized grip strength (more affected side as a percentage of less affected side) was 30.5 ± 0.20%. Three patients had cortical and 12 had subcortical stroke. Nine patients had ischemic and six patients had hemorrhagic stroke. All strokes occurred in the left middle cerebral artery territory. The time since stroke onset was 42.7 ± 40 months (median 82 months, inter-quartile range 30–145 months). All patients were right-handed before the infarct and hence had hemiparesis affecting their dominant side.

## Index of elbow extension (IoEE)

**Fig. 1:**
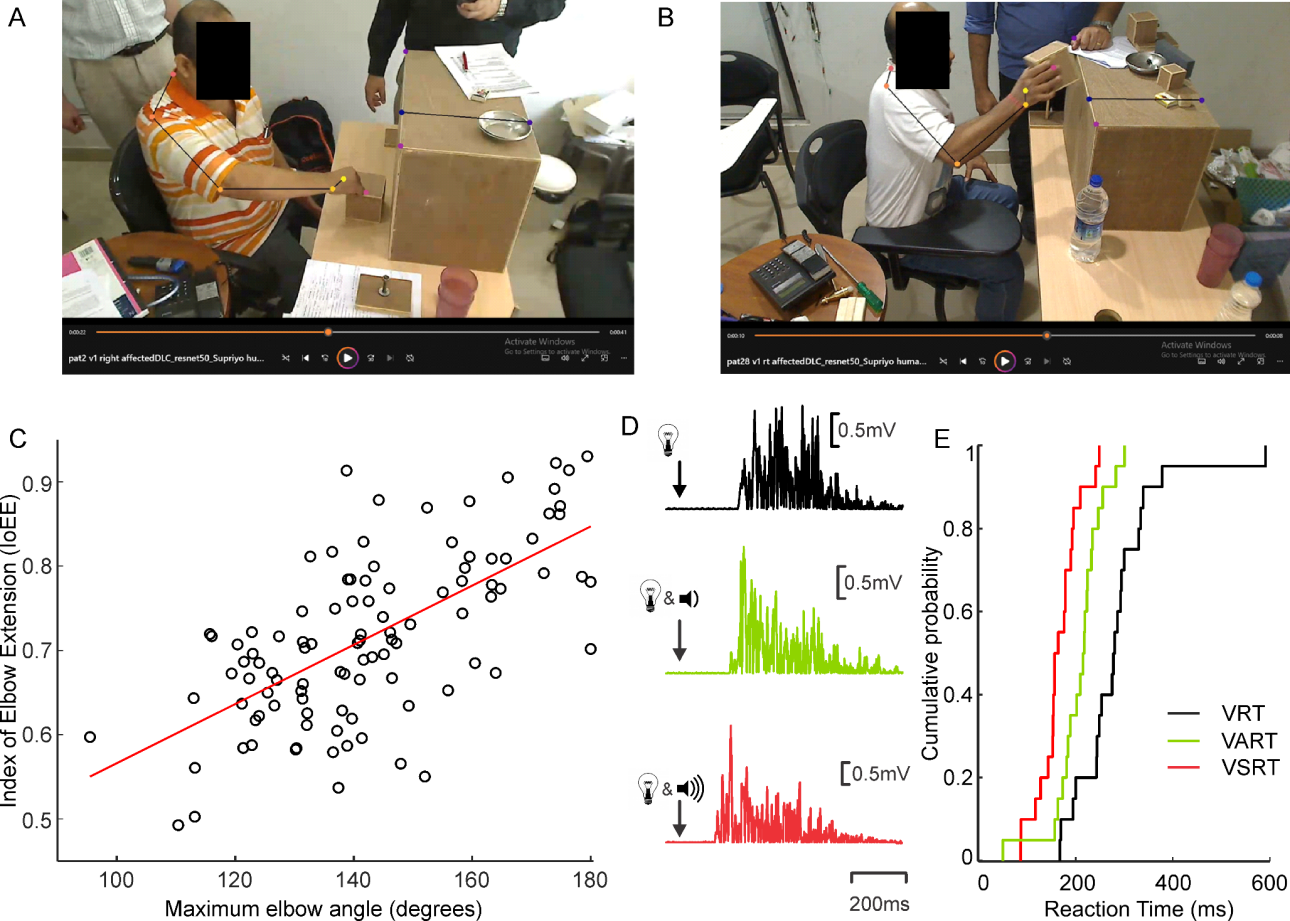
Example data. **A**, Annotated video frame with bony landmarks assigned using DeepLabCut (DLC) and skeleton connecting the landmarks in a patient who could reach but could not grasp the object. **B**, DLC annotated video frame acquired from a patient who could grasp the object and reach to place it on the shelf. **C**, scatter plot of Index of Elbow Extension (IoEE, determined from 2D video) versus maximum elbow angle (determined from 3D measurements), in healthy subjects asked to mimic movements from the stroke cohort. Red line shows the linear regression fit. **D**, rectified EMG acquired during a Visual Reaction Time (VRT; black trace), a Visual Auditory Reaction Time (VART; green trace) and a Visual Startle Reaction Time (VSRT; red trace). **E**, VRT, VART and VSRT results from a single patient summarized as cumulative probability distributions of the reaction times showing clear separation between responses to the three cues (VRT > VART > VSRT)

Figure 1A and B illustrate frames from the videos for reaching attempts with varying success by two different patients. The first patient could reach the object but failed to grasp it, whereas the second patient could grasp the object successfully and reach to place it on the shelf in front of them. Superimposed on the frame are markers determined by DeepLabCut.

The IoEE is a measure made from a standard video, which was designed to relate approximately to the maximum elbow angle achieved during a reaching movement. In order to validate this novel measure, we made recordings using a specialist hybrid camera capable of 3D measurements. Healthy participants were asked to mimic the reaching behavior seen in videos of stroke survivors; this allowed us to collect a large number of videos of diverse reaching examples in a short time. Figure 1C presents a scatter plot of the IoEE (measured from a conventional color video feed) versus the gold standard maximum elbow angle, measured from 3D data on shoulder, elbow and wrist positions. The two measures had a correlation coefficient of *r* = 0.65 (*P* < 0.001), indicating that the IoEE is a reasonable proxy for maximum elbow angle.

## StartReact

Figure 1D illustrates three trials of EMG activity from forearm flexor muscles in response to the different cues. The reaction time to a visual cue alone (VRT, black) was longer than to a quite sound combined with a visual cue (VART, green), which in turn was longer than to a loud sound with visual cue (VSRT, red). Figure 1E presents the reaction times to these cues in a single stroke survivor as cumulative probability distributions, which confirms the differences in latency seen in the single sweeps. Across the population of stroke survivors studied here, the reaction times were: VRT, 326 ± 96ms; VART, 274 ± 89ms; VSRT, 225 ± 80ms. The StartReact effect (VSRT-VART) was 50 ± 42ms, compared with 45 ± 25ms observed in healthy volunteers.

**Fig. 2:**
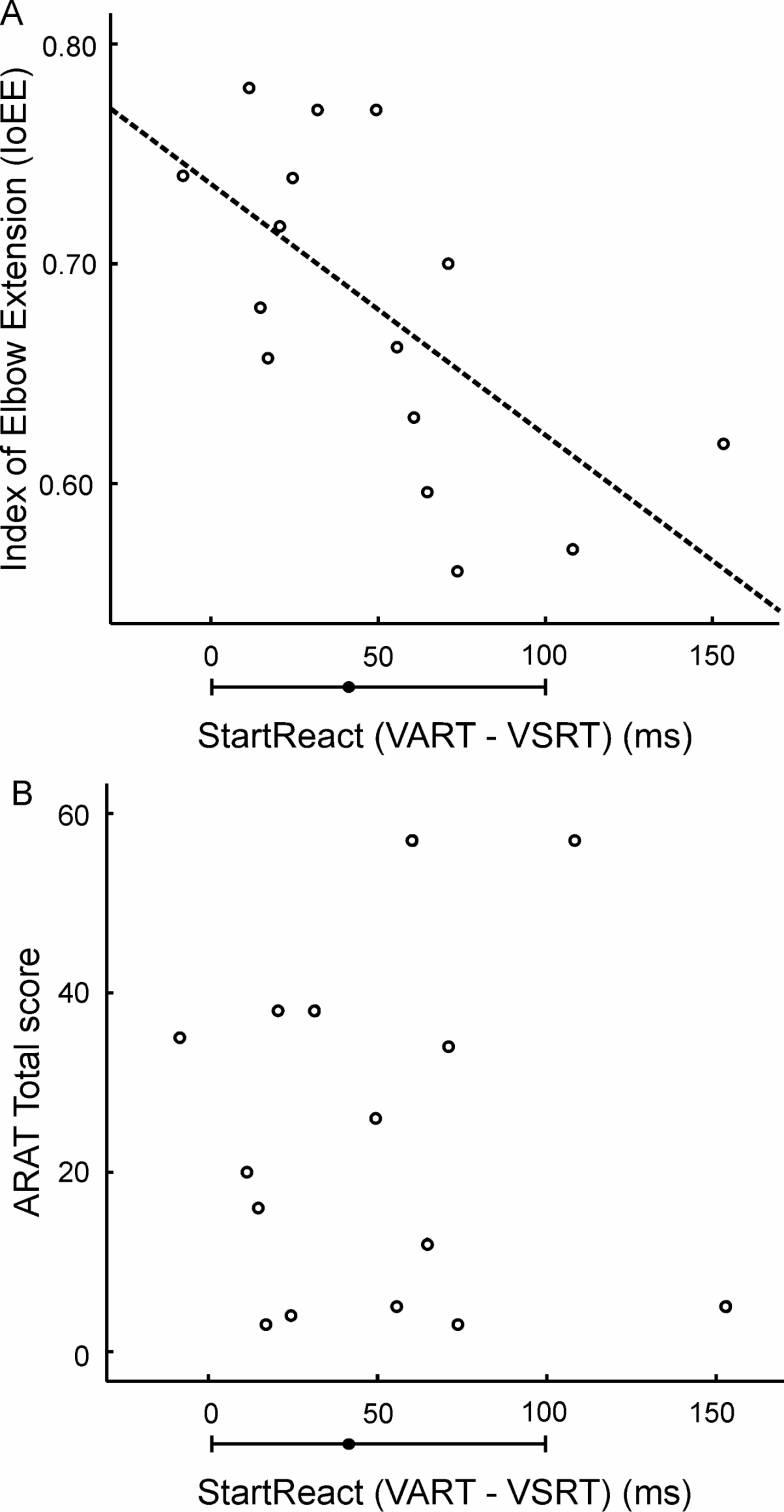
Correlations with StartReact. **A**, scatter plot between Index of Elbow Extension (IoEE) and StartReact (VART-VSRT); dotted line shows the linear regression fit. **B**, scatter plot between ARAT total score and StartReact. The horizontal error bar below the abscissa indicates the median and 95% range of VART-VSRT in healthy individuals

Table 1 presents the correlation found between the IoEE and various clinical and reaction time measures. Although IoEE was not related to measures of reaction time to particular cues (VRT, VART, VSRT), it was significantly correlated with the StartReact effect (VART-VSRT; Fig. 2A), which has been shown to relate to the strength of reticulospinal connections (Tapia et al. 2022). None of the clinical measures had a significant correlation with IoEE. In our previous study using a larger sample size, the ARAT score was shown to correlate negatively with the StartReact effect. This correlation was not observed in the present smaller sample (Fig. 2B; rho=-0.016, *p* = 0.96).

**Table 1:** Correlation of various clinical and reaction time measures with the index of elbow extension (IoEE), over 15 stroke survivors. Correlation is given as the spearman rank correlation coefficient Rho, and its associated uncorrected P value. *, *p* < 0.05 after correction for multiple comparisons

Correlation with Index of Elbow Extension (IoEE; *n* = 15)	Rho	*p*
Modified Ashworth Score (MAS) for Forearm Flexors	-0.409	0.130
Total ARAT score of affected upper limb	0.236	0.397
ARAT grasp sub-score of affected upper limb	0.269	0.332
ARAT grip sub-score of affected upper limb	0.182	0.516
ARAT pinch sub-score of affected upper limb	0.148	0.600
ARAT gross sub-score of affected upper limb	0.171	0.543
Normalised hand Grip of most affected side as percentage of less affected side	-0.052	0.854
Visual Reaction Time (VRT)	-0.413	0.126
Visual-Auditory Reaction Time (VART)	-0.424	0.116
Visual-Startle Reaction Time (VSRT)	0.102	0.718
StartReact (VART-VSRT)	-0.702*	0.004

## Discussion

Here we provide further evidence on the relationship between the strength of reticulospinal connections mediating upper limb function and recovery of reaching after hemiparetic stroke in humans. Additionally, we tested the utility of the open-source deep learning-based computer vision software DeepLabCut in the field of clinical neuroscience.

In the current study we found evidence that StartReact (an indirect marker of RST activity) was negatively correlated with the IoEE. This suggests that RST connection strength is greater in patients where recovery of reaching is incomplete. We think it most likely this correlation arises from correlation with a third (unmeasured) variable, the extent of stroke-related damage to the corticospinal tract (CST). Patients with more damage would have more restricted reaching, and also rely more on compensatory activation of RST pathways. This would explain the reverse correlation. However, an alternative hypothesis could be that increased engagement of RST pathways produced flexor spasticity, interfering with elbow extension and thus restricting reaching. However, against this idea, the modified Ashworth Score assessing hypertonia at the wrist showed no correlation with the IoEE, suggesting that these two attributes are independent aspects of post-stroke motor recovery.

Surprisingly, in this cohort we did not find any correlation between the ARAT score, MAS score or grip strength with the IoEE. In our previous study (with three times the number of patients, of which the present group was a sub-set) the ARAT showed a correlation with StartReact (Choudhury et al. 2019). Contrary to our current findings, another group found that the extent of elbow extension was the largest contributor to the variance in the Fugl-Meyer assessment and ARAT, together with grip strength (Nijenhuis et al. 2018). The unexpected null finding in our study possibly therefore indicates that the ARAT score depends on multiple attributes of upper limb function and not just the extent of reaching.

Rahimi and Honeycutt (2020) tested whether a loud sound applied at the same time as a go cue could enhance reaching in stroke survivors. In all individuals, startle trials had a larger amplitude of muscle activation and faster EMG onset, although the effect was considerably larger for severely affected subjects. This led to severely affected participants producing larger reaching distances after a startling cue, but reaching was not always accurately directed towards the cued target. In part inaccurate reaching was due to excessive activation of flexors, even when the cued reach required extension (also reported by Honeycutt and Perreault, 2012). In the present study, we showed that the estimated RST contribution to muscle activation was larger in individuals with worse impairment (assessed by reduced elbow extension during reaching). The more inputs from the RST are strengthened, the more we would expect that a startle sound which enhances RST activity (Tapia et al. 2022) would increase muscle activity. The finding by Rahimi and Honeycutt (2020) that startle had a greater effect on muscle activation and reaching in people with severe impairment is thus consistent with our results. The propensity of strengthened RST connections after a CST lesion to activate flexors rather than extensors has been reported previously in monkey (Zaaimi et al. 2012), as well as by Rahimi and Honeycutt (2020). Excessive activity in flexors could lead to reduced elbow extension during reaching, possibly explaining the negative correlation between RST contribution and the IoEE which we observed (Fig. 2A).

This study shows that useful kinematic measures of functional movement can be extracted from web cam video, in combination with marker less motion tracking software (DeepLabCut). Previous studies have assessed performance using more complex equipment, in which the patient’s trunk was restrained (McPherson et al. 2018). In such a configuration, reaching performance can be measured by the area swept out by the subject’s hand when reaching as far from the body as possible. With a restrained trunk, limitations of reach extent correspond to an inability to extend at the elbow and shoulder. In our configuration, the trunk was free to move. This allowed subjects to choose whether to complete the task with the body nearly vertical (as in healthy subjects), or to lean forward to compensate for limited elbow extension. However, our index directly measured elbow extension. In hemiparetic patients, it thus probably provides a very similar metric to reach extent with a restrained trunk. The only potential limitation is if there is learned non-use. This occurs where a subject learns that some movements are not possible in the immediate aftermath of a stroke. Even though there may be subsequent recovery, these movements are not then explored. This could occur, even if subjects are encouraged to reach without leaning forwards as here. In such cases, the IoEE in the current experimental arrangement would underestimate the possible extent of elbow extension. Nevertheless, the IoEE has been shown to be a useful and readily-applicable measure. The ability to use low-cost tools to deliver quantitative kinematic metrics may allow larger scale deployment of such methods, either in routine clinical care or as adjuncts to clinical rating scales such as ARAT.

## Data Availability

The data that support the findings of this study are available on request from the corresponding authors. The data are not publicly available due to their containing information that could compromise the privacy of research participants.

## Abbreviations

RST: Reticulospinal tract
ARAT: Action Research Arm Test
PMRF: Pontomedullary reticular formation
VRT: Visual Reaction Time
VART: Visual Auditory Reaction Time
VSRT: Visual Startle Reaction Time
IoEE: Index of Elbow Extension
CST: Corticospinal tract

## Declarations

### Statistical analysis

Prof. Stuart N Baker, Dr Supriyo Choudhury.
